# Modalities for Imaging of Prostate Cancer

**DOI:** 10.1155/2009/818065

**Published:** 2010-03-17

**Authors:** A. H. Hou, D. Swanson, A. B. Barqawi

**Affiliations:** Division of Urology, University of Colorado Health Sciences Center, UCDSOM, MS C-319, Academic Office One Bldg., 12631 East 17th Ave., Room L15-5602, Aurora, CO 80045, USA

## Abstract

Prostate cancer is the second most common cause of cancer deaths among males in the United States. Prostate screening by digital rectal examination and prostate-specific antigen has shifted the diagnosis of prostate cancer to lower grade, organ confined disease, adding to overdetection and overtreatment of prostate cancer. The new challenge is in differentiating clinically relevant tumors from ones that may otherwise never have become evident if not for screening. The rapid evolution of imaging modalities and the synthesis of anatomic, functional, and molecular data allow for improved detection and characterization of prostate cancer. However, the appropriate use of imaging is difficult to define, as many controversial studies regarding each of the modalities and their utilities can be found in the literature. Clinical practice patterns have been slow to adopt many of these advances as a result. This review discusses the more established imaging techniques, including Ultrasonography, Magnetic Resonance Imaging, MR Spectroscopy, Computed Tomography, and Positron Emission Tomography. We also review several promising techniques on the horizon, including Dynamic Contrast-Enhanced MRI, Diffuse-Weighted Imaging, Superparamagnetic Nanoparticles, and Radionuclide Scintigraphy.

## 1. Introduction

Prostate cancer is the second most common cause of cancer deaths among males in the United States. The incidence of prostate cancer is relatively constant at 165 cases per 100,000 men. Since 1990, the age-adjusted death rate has progressively decreased by 31%, which is attributed to early detection and treatment [1]. Prostate screening by digital rectal examination (DRE) and prostate-specific antigen (PSA) has shifted the diagnosis of prostate cancer to lower grade, organ confined disease [2, 3], adding to overdetection and overtreatment of prostate cancer by at least 30% [4]. A recent review by Etzioni et al. estimated that 10% of men with low-grade prostate cancer were overtreated with radical surgery, and 45% were overtreated with radiation therapy [5]. With the publication of the 10-year results of the Prostate, Lung, Colorectal, and Ovarian (PLCO) Cancer Screening Trial demonstrating no reduction of mortality with screening, the new challenge is in differentiating clinically relevant tumors from ones that may otherwise never have become evident if not for screening [6].

The rapid evolution of imaging modalities allows for better detection and staging of prostate cancer, thus directing appropriate treatment and follow-up. However, the appropriate use of imaging is difficult to define, as many controversial studies regarding each of the modalities and their utilities can be found in the literature. Here, we will discuss the more established imaging techniques, and will review several promising techniques on the horizon. 

## 2. Transrectal Ultrasound

### 2.1. Grey-Scale Ultrasound

Grey-scale transrectal ultrasound (TRUS) is the most commonly used modality for evaluating the prostate, particularly for guiding needle biopsies. When prostate cancer is suspected due to an elevated PSA or an abnormal digital rectal exam (DRE), the next step is usually a systematic needle biopsy, for which TRUS is integral and effective in identifying the outlines of the prostate in sagittal and transverse planes. It is a simple and readily available modality, and can provide fairly accurate estimations of the prostate volume which are important in the determination of PSA density [7]. Prostate cancers typically appear hypoechoic on TRUS [8], and hypoechoic lesions on TRUS are more than twice as likely to contain cancer as isoechoic areas [9]. However, most hypoechoic lesions found on TRUS are not cancer [10]. Moreover, up to 30% of prostate cancers are isoechoic [11], and approximately 1% are hyperechoic [8]. The positive predictive value (PPV) of grey-scale TRUS is reported to be 52.7%, and the negative predictive value (NPV) is 72%, with an accuracy of 67% [12]. Thus, its utility in the detection and localization of prostate cancer is somewhat limited. However, it remains the imaging modality of choice for guided prostate biopsies, as well as for directing brachtherapy, cryotherapy, high-intensity focal ultrasound ablation, and other locally-directed therapies including hyperthermia, photodynamic therapy, tumor vaccines, and gene therapy [13].

The value of TRUS for local staging is controversial, although several studies have established criteria for distinguishing extracapsular extension (ECE) on TRUS, including bulging or irregularity of the capsule adjacent to a hypoechoic lesion [13], as well as length of contact of a lesion with the capsule [14]. A multiinstitutional study found TRUS to be no more accurate than DRE for the purpose of detecting local tumor extension [15], although other early reports found an increased accuracy when ultrasound findings were combined with DRE and PSA level [16]. Other studies have also found poor pathologic correlation and inaccurate prediction of clinical stage with TRUS and have shown that impalpable tumors have similar outcomes regardless of TRUS findings [17, 18]. However, a more recent study of 620 men who underwent TRUS by a single ultrasonographer prior to radical prostatectomy found that evidence of extra-capsular extension (ECE) by TRUS correlated with a significantly higher pathologic stage [19]. They found that TRUS staging was the most significant predictor of ECE compared with established risk factors such as Gleason grade and serum PSA, and furthermore that TRUS staging performed as well as these other variables in predicting biochemical failure after radical prostatectomy. This study was performed at a single institution by a single highly experienced ultrasonographer, and has yet to be replicated in other reports.

### 2.2. Contrast-Enhanced Ultrasound

Interest in increasing the sensitivity of ultrasound has led to the development of multiple techniques based on ultrasound but incorporating additional technology. Contrast enhanced TRUS (CEUS) combines the value of traditional ultrasound in spatial and temporal visualization of the prostate with the observation that the process of prostatic tumor growth induces neovascularization [20]. CEUS is a method of measuring the intraprostatic vascular structures using microbubbles as the contrast agent. 1–10 *μ*m gas-encapsulating spheres (microbubbles) are injected into the bloodstream, and then visualized as they flow through the prostate using a transrectal ultrasound probe. The bubbles function as additional reflectors to increase the sensitivity of color and power Doppler [21]. Abnormal findings on CEUS have been found to correlate with cancer on pathologic examination [22].

In the early days of CEUS, when high-energy ultrasonography was used, the majority of the microbubbles were destroyed by the pulsations, hindering visualization of the microvasculature [23]. In recent years, several developments in the technology have led to increased sensitivity of microbubble visualization in the microvasculature of the prostate. Wink et al. recently evaluated the results of multiple European studies which correlated CEUS and histology findings on prostate resection, and concluded that tumor localization using CEUS is a promising technique. The data supports the use of CEUS in aiding in visualization of prostate cancer, but the sensitivity and specificity are not high enough to justify discontinuing systematic prostate biopsies [21].

Using contrast enhanced-ultrasound to target biopsies has been found to detect statistically significantly more cancers with fewer number of cores compared to traditional gray-scale ultrasound-guided biopsy [24]. The same study found a 2.6 fold higher likelihood of detecting prostate cancer when contrast-enhanced ultrasound was used, over gray-scale ultrasonography. A study from the Netherlands [25] examined how well was three-dimensional color-enhanced power Doppler ultrasound (3D-CE-PDU)-directed prostate biopsies compared with radical prostatectomy specimens. All patients in the study were known to have prostate cancer and were already scheduled for radical prostatectomy. Overall, 51% to 63% of the prostate cancers were detected by contrast-enhanced ultrasound. There was a higher sensitivity (68%–79%) for detecting cancers with a maximum diameter of ≥5 mm. Of note, the study found only 13% of extracapsular extension or seminal vesicle invasion, demonstrating that 3D-CE-PDU is a poor tool for staging.

### 2.3. Elastography

Another approach based on TRUS is elastography, which measures the rigidity and elastic properties of the prostate. The prostate is compressed using the transrectal probe, and the differences in tissue strain are used to localize intraprostatic lesions. Similar to the digital rectal exam, areas of increased firmness are more suspicious for malignancy. Pallwein et al. [26] found a sensitivity and specificity of 87% and 92% for detecting prostate cancer foci in a study of 15 patients who underwent real-time elastography with subsequent radical prostatectomies. They detected 28 of 35 tumor foci, with highest sensitivity (100%) for detecting cancer at the apex. In another study of 137 men who underwent targeted prostate biopsies with elastography with color Doppler, an odds ratio of 1.82 was found, with a significantly higher likelihood of cancer detection than with gray-scale sextant biopsy [27]. A greater than two-fold increased likelihood of malignancy was noted in association with ultrasonic abnormality; abnormal color flow was associated with Gleason 8–10 (OR 4.12–10.61). This association was not found in lower-grade lesions with color Doppler. However, abnormal elastographic findings were associated with malignancy for all tumor grades.

In 2008, Salomon et al. [28] reported on a prospective single institution study of 109 men who were scheduled for radical prostatectomy and underwent ultrasound-based elastography-directed prostate biopsy. The PPV between suspicious areas on elastography and cancerous areas found on pathologic examination after radical prostatectomy was 87.8%, and the NPV was 59%. The sensitivity and specificity of elastography in detecting cancer were 75.4% and 76.7% respectively. Specificity increased to 80% when the lesion was ≥5 mm in diameter. Another study of 107 men with PSA > 4 ng/mL or abnormal digital rectal examinations who underwent elastographic studies, regular transrectal ultrasound, and power Doppler ultrasonography, found the sensitivity of elastrography to be similar to that of power Doppler (68% versus 70%) and higher than regular ultrasound (50%), with a specificity of 81% [29]. Overall, elastography seems to be a feasible, reproducible tool with fairly good sensitivity for detecting prostate cancer. Further clinical studies to clarify the role of elastography in the detection of prostate cancer and its use in guiding prostate biopsies are ongoing.

### 2.4. Computer-Aided Ultrasonography

Computer-aided ultrasonography, also known as HistoScanning, allows mapping of prostatic morphology, and identification of malignant lesions by characterizing and quantifying the disorganization of the tissue. Theoretically, malignancy will induce disorganization, and computer-aided ultrasonography will detect these changes by extracting this data from background ultrasonographic data. In a study of 29 patients who underwent radical prostatectomy after Histoscanning, a close correlation between prostate HistoScanning analysis and pathologic findings was found. The test was capable of localizing tumors and determining their cross-sectional size with fairly good accuracy, as well as identifying multifocality, bilaterality, and extraprostatic extension. The authors of the study propose that HistoScanning is an inexpensive, noninvasive test with reasonable accuracy that could further guide clinical decision making when it comes to selecting men with high PSAs to undergo prostate biopsy [30]. However, further studies to validate this data are lacking.

## 3. Magnetic Resonance Imaging

### 3.1. T2-Weighted Magnetic Resonance Imaging

The role of standard T2-weighted MRI in the diagnosis and staging of prostate cancer is still evolving and varies from center to center. Generally, MRI has a fairly good sensitivity but poor specificity [31–33]. A recent meta-analysis of studies correlating MRI and histopathology found reported sensitivities between 37% and 96% for detecting prostate cancer, with differences due to variable definitions of cancer, exclusion of transitional zone cancers, and criteria used for positive findings [34]. Specificity ranged from 21% to 67% in this meta-analysis, although the authors note that specificity is difficult to assess due to inherent group selection bias in these studies.

The specificity of MRI is decreased by its inability to distinguish the low T2 signal intensity that is associated with tumor from other pathologies such as prostatitis, postbiopsy hemorrhage, or treatment changes [35]. Generally, a delay of three to four weeks between prostate biopsy and MRI was recommended [36, 37], although a more recent study recommended a delay of six to eight weeks when more biopsy cores are taken due to a higher degree of hemorrhage [38].

Detection of prostate cancer in the transitional zone was thought to be inferior. A study by Ellis et al. found that MRI missed 78 of 79 tumors in the anterior gland that were >5 mL in volume, although this study was done without the use of endorectal MRI [39]. Other studies have shown that MRI has roughly equal accuracy throughout the prostate [35], and that MRI has good sensitivity and specificity (75% and 87%) in detecting transitional-zone cancers as well [40]. Akin et al. [40] suggested that several indicators of transitional zone cancer include homogenous low signal, lenticular shape, and invasion of the anterior fibromuscular stroma.

#### 3.1.1. Screening

Many possible applications of MRI have been proposed and studied. Some argue that it can contribute to the screening process. In patients with a positive PSA screening test but a negative biopsy, a negative MRI result may eliminate the need for repeat biopsy by increasing the NPV of a negative prostate biopsy. In a screening population of 92 patients, Comet-Batlle et al. demonstrated an NPV for cancer on subsequent transrectal biopsy of 91% [41], which is comparable to the 85% NPV of a negative octant biopsy [42]. Cheikh et al. reported an 82.6% sensitivity and 100% NPV of T2-weighted or dynamic contrast-enhanced MRI when evaluating visible suspicious areas prior to repeat TRUS prostate needle biopsy [43]. Beyersdroff et al. studied a population of 44 men who had elevated PSA but negative initial biopsy, and found that in retrospective analysis of MRI after a repeat biopsy was performed, MRI imaging results did not correlate with biopsy findings [31]. To the authors, this confirmed that MRI has a fairly low specificity and is subject to error due to prostatitis, fibrosis, or intraepithelial neoplasia. The widespread use of MRI for screening purposes has not been implemented despite several promising studies, largely due to the high costs, although its use in selected patients may be warranted after negative prostate biopsies.

#### 3.1.2. MRI-Guided Prostate Biopsy

The use of MRI to guide prostate biopsies has also been proposed and is currently under investigation and development. Beyersdroff et al. published reports of successful MRI-guided prostate biopsy, noting that the technique was time-consuming and required specific equipment, and was very susceptible to prostate movement during biopsy [44]. Multiple groups are experimenting with needle-positioning devises and robotic manipulators to facilitate MRI-guided biopsies [45]. However, routine use of MRI to guide prostate biopsy is not recommended with existing evidence, due to expense and the time-consuming nature; although with further developments and experience, there may be indications for MRI-guided biopsies in patients with previous negative TRUS-guided biopsies.

#### 3.1.3. Preoperative Assessment

Recently, MRI has been evaluated for its utility in preoperative assessment prior to prostatectomy. Hricak et al. found that by reviewing preoperative endorectal MR images, surgeons were more accurately able to decide whether or not to preserve the neurovascular bundle during surgery [46]. 135 patients were evaluated with MRI preoperatively and judged by a surgeon and radiologist, then compared to surgeon's clinical judgement during surgery and to histopathologic findings. They found that when the surgeon decided to spare the NVB, MRI confirmed that decision in 84% of cases, and was correct in 96% of the time. MRI results changed the surgical plan in 78% of high-risk patients (>75% risk of ECE on Partin tables), and was correct in 93% of cases. These results were supported by a recent study from Scandanavia of 75 patients, which showed a sensitivity of 92% and specificity of 100% for detection of ECE/NVB involvement with preoperative MRI [47]. MRI findings favored NVB preservation in 67% of patients with a high-clinical probability of ECE, and opposed NVB preservation in 33% of patients with low probability, and was correct in 100% of the time. Another series showed that prominence of apical periprostatic veins on preoperative endorectal MRI was significantly associated with intraoperative blood loss [48]. These studies support the use of MRI for preoperatively assessing patients, particularly regarding the decision to spare or resect the neurovascular bundles.

#### 3.1.4. Staging

Due to many advances in technology, MRI is now considered by many to be the most exact imaging modality for staging prostate cancer, including pelvic lymph node and pelvic bony metastases [49–53], although studies have found that there is significant intraobserver variability in the use of MRI for detecting prostate cancer [54]. Several early studies found that the sensitivity of MRI in detecting extra-capsular extension was limited [55–57]; however, developments in technology have improved the accuracy for local staging. The use of endorectal-body phased array coils, as opposed to a torso phases-array coils, has been shown to offer superior staging accuracy [58]. This technique improves the spatial resolution and signal-to-noise ratio of prostate MRI [31]. Endorectal MRI has been found to have high negative and positive predictive values, as well as good accuracy in predicting extracapsular invasion [58, 59]. Two meta-analyses of staging accuracy with endorectal MRI have been conducted, with differing results; one found that endorectal MRI improved staging performance [60], whereas the other found the opposite [61].

Other recent developments have improved the accuracy of T2-weighted MRI in local staging as well, including faster imaging sequences, postprocessing image correction, and more powerful coils [58, 62, 63]. The use of persextant localization is another development in MRI technology that has allowed more efficient communication of data [35, 64]. Theoretically, a 3Tesla endorectal coil should provide improved signal-to-noise ratio and improve image quality, and several early reports do report significantly improved staging performance, with sensitivities of 73–88% and specificities of 96–100% [65, 66]. However, this technology is not widely available, and more studies are needed before widespread implementation.

Despite these technological advances, the use of MRI for staging prostate cancer is still fairly controversial, and its use at different institutions is still evolving. Several studies have focused on the utility of MRI across different risk groups in an attempt to solve the issue of which patient population should undergo preoperative MRI for staging. It has been found that the inclusion of MRI in clinical nomograms increases the prediction of local tumor extent in all risk groups [67]. Engelbrecht et al. have suggested that in patients with an intermediate risk of having stage T3 disease, as predicted by having a PSA of 4–20 ng/mL and a Gleason score between 5 and 7, MRI is advised because the treatment decision may depend on imaging results [68]. Another group found that 26% of low-risk patients, with PSA < 10 ng/mL and Gleason 2–7 or PSA 10.1–20 ng/mL and Gleason 2–5, had histopathologic proof of ECE or SVI seen on MRI prior to surgery [63], which may indicate justification of routine MRI in this group as well. Furthermore, The American Joint Commission on Cancer currently recommends either CT or MRI in the high-risk group, with PSA > 20 or Gleason > 7-8 [69].

Those who argue against its routine use in preoperative evaluation cite its expense and questionable utility. D'Amico et al. suggested that although MRI does increase the prediction of biochemical failure in a significant number of patients, the high cost of routine use of MRI is not justified in light of questionable effect on decreasing unnecessary surgeries [70]. Another study by May et al. evaluating at the ability of endorectal MRI to detect and stage prostate cancer concluded that due to poor sensitivity and specificity, high intraobserver variability, and a tendency to overstage cancer, MRI results should not result in alteration of treatment decisions [71].

### 3.2. Magnetic Resonance Spectroscopic Imaging

Intraprostatic tumor growth is associated with increased cell membrane turnover and increased cell proliferation, which lead to altered relative concentrations of certain metabolites including creatine, choline, and citrate, most specifically an increase in choline and a decrease in citrate. Magnetic resonance spectroscopy (MRSI) is a technology that increases the sensitivity and specificity of MRI by analyzing this metabolic profile of discreet voxels within the prostate [72, 73]. The largest study comparing MRSI to MRI, by Wefer et al., found that MRSI alone had a higher sensitivity (76%) than T2-weighted MRI (67%), but a lower specificity. However, this and other studies have found that the accuracy in diagnosing prostate cancer is the highest when anatomic information from MRI and metabolic information from MRSI are combined [35, 72, 74]. Another study combined data from MRI/MRSI with clinical and pathological data, and found that a combined model was superior to purely clinical models at predicting the probability of insignificant prostate cancer [75].

More recently, several studies have analyzed the effects of adding data from MRI/MRSI to the staging normograms. In a study of 229 patients who underwent MRI and 383 who underwent combined MRI/MRSI prior to radical prostatectomy, the radiologic findings contributed significant value to the standard normogram for predicting prostate-confined disease [67].

Spectroscopy has several other advantages over traditional MRI. The ability of MRSI to detect transition zone tumors has been reported to be 80%, significantly higher than T2-weighted MRI [40]. Coakley et al. [76] explored the ability of MRSI to estimate the volume of prostate cancer, and found improved accuracy in volume measurements when spectroscopy is added to MRI. Yu et al. also demonstrated that use of spectroscopic imaging decreases interobserver variability, and for less experienced radiologists, improved the detection of ECE [49].

A recent multiinstitutional study of 134 men with biopsy-proven prostate cancer who underwent combined MRSI/MRI found somewhat disheartening results [77]. When compared to endorectal MRI, combined MRSI/MRI showed no demonstrable benefit. This study was specifically conducted with inexperienced operators, and was focused on low-risk patients with small disease burden, representative of the screened American population.

#### 3.2.1. Tumor Aggressiveness

MRSI has potential as a noninvasive method of assessing tumor aggressiveness, as it has a higher sensitivity in detecting cancers of higher grade [78]. Zakian et al. studied the relationship between Gleason grade as a measure of tumor aggressiveness and MRSI volumetric and metabolic data, and found that the ratio of creatine plus choline to citrate, which is a ratio that is known to be positively correlated with prostate cancer, can predict the aggressiveness of the tumor. This study also found that MRSI detected only 44% of Gleason 3 + 3 tumors, compared to 90% of Gleason >7 cancers. Thus, spectroscopic information may contribute to the decision-making process by providing information about tumor aggressiveness.

#### 3.2.2. Additional Uses

Additional uses of MRSI in prostate cancer have been proposed, including supplementing biopsies as a method of detecting both primary and recurrent cancers [34, 35], and as a tool to guide biopsies [79, 80]. Due to the higher sensitivity of MRSI in detecting cancer than sextant biopsies, especially when localized to the apex where biopsies may not sample [35], it may be an adequate method of following patients who have undergone ablative therapy. One study found that MRSI detected all recurrent foci of tumor in 25 patients who had undergone cryotherapy for prostate cancer [81]. Another study of 9 patients with recurrent prostate cancer after external beam radiation therapy found that MRSI and MRI were both superior to biopsy at detecting recurrent disease, with sensitivities of 77% and 68%, respectively [79]. However, they also found that the specificity of MRSI (78%) was significantly lower than regular MRI, DRE, or sextant biopsy, which was attributed to postradiation metabolic changes in the normal prostate. The authors suggested that MRSI may be used to supplement other techniques to detect recurrent cancer, to guide biopsies, or to guide treatment by providing localization of tumor [79].

### 3.3. Dynamic Contrast-Enhanced MRI

Dynamic contrast-enhanced-MRI (DCE-MRI) measures the vascularity of prostatic tissue using temporal differences in the uptake of intravenously-administered low molecular weight contrast to distinguish between benign tissue and tumor. Increased microvessel density is seen in BPH, prostatic intraepithelial neoplasia, and prostate cancer [82], and has been found to correlate with disease-specific survival and progression after treatment [83]. Several microvascular features are characteristics of prostate cancer, including heterogenic structure, arterio-venous shunting, vascular tortuosity, intermittent flow, high permeability, and poorly formed vessels [82]. Using various MR sequences, especially T1-weighting [84], these characteristics can be visualized and quantified.

The use of DCE-MRI for primary detection, localization, and staging of prostate cancer has been studied by several groups, with promising results. Jager et al. initially found that the use of DCE-MRI increased the sensitivity for detecting prostate from 57% with T2-weighted MRI to 73%, with no change in specificity [85]. They also noted that DCE-MRI may improve sensitivity for detecting ECE, and that this technology may improve estimations of tumor volume. Other studies have confirmed that DCE adds significantly to the sensitivity for detecting prostate cancer over other modalities [86–89] and that it is fairly accurate in detecting ECE [90, 91]. A recent prospective study found that DCE-MRI had higher localization accuracy than T2-weighted MRI or MRSI in both the central and peripheral glands [92]. When compared to transrectal power-dopper ultrasound, DCE-MRI was found to be significantly more sensitive for detecting cancer in the peripheral zone [89]. Namimoto et al. confirmed that DCE-MRI is useful in the differentiation of peripheral-zone lesions, and also found higher diagnostic accuracy when dynamic MR results were combined with postcontrast T1-weighted images [93]. Other proposed uses for DCE-MRI that include detection of recurrence and follow-up after ablative therapy or androgen deprivation [82].

There is a potential for DCE to predict pathologic grade of prostate cancer, given that microvessel density has been found to correlate with Gleason score [94, 95]. Schlemmer et al. noted that time to onset of the enhancement curve was significantly different in high-grade tumors compared to low-grade tumors [86]. Another study using pathologic specimens for correlation did not find any correlation between enhancement times and pathologic grade [96].

Kim and Park speculated that DCE-MRI may benefit immensely from the advent and institution of 3 Tesla due to increased signal-to-noise ratio and because of the T1 properties of prostate tissue [97]. An early study found that at 3 Tesla, DCE-MRI has a higher accuracy and sensitivity, although a lower specificity, than T2-weighted imagining, and concluded that DCE-MRI may thus be more useful for diagnosis and preoperative staging [51]. Fütterer et al. found that DCE-MRI at 3 Tesla was superior to 1.5 Tesla in regards to delineation of prostate cancer, extra-capsular extension, and visualization of the peripheral zone and central zone [98]. They found an overall staging accuracy of 83% at 1.5 Tesla, and 100% at 3 Tesla.

It is clear that more studies are needed the define to role of DCE-MRI in the evaluation of prostate cancer. Major limitations of this technology include the inability to differentiate between prostatitis and prostate cancer in the peripheral zone and between BPH and cancer in the central zone [99]. With current evidence, it has been proposed that DCE-MRI can be used in combination with T2-weighted MRI, MRSI, and DWI as part of an assessment of cancer probability, to guide targeted rebiopsy [82], and to preoperatively stage patients with prostate cancer [51, 91].

### 3.4. Diffusion-Weighted Imaging MRI

Diffusion-weighted imaging (DWI) is a method of obtaining molecular and cellular information about the prostate, specifically regarding the movement and functional environment of water in prostate tissue. By measuring the microdiffusion of water in the intracellular and extracellular spaces, DWI can calculate an apparent diffusion coefficient, which reflects compartmental shifts in water, membrane permeability, and cellular density, all of which may be altered in cancerous tissue [100]. Prostate cancer has a lower apparent diffusion coefficient than does normal prostate tissue, both in the peripheral zone [101] and in the transition zone [102], which indicates a reduction in flow or diffusion of water. This is attributed primarily to an increase in cellularity in this tissue [101, 102].

Several studies have analyzed the value of adding DWI to T2-weighted MRI and MRSI, and have generally found increased sensitivity (54%–98%) and specificity (58%–100%) [100, 103, 104]. Kim et al. recently found that combined DWI/T2WI is more sensitive for predicting recurrent cancer after radiation therapy than T2WI alone [105]. DWI has also been found to increase the accuracy of peripheral zone tumor volume measurements [106]. Park et al. recently reported that DWI may be useful in the evaluation of patients with persistently elevated PSA values but negative prostate biopsies, as it was more sensitive than T2-weighted MRI in localizing lesions [107].

Major limitations of this technology include a substantial overlap of ADC values between malignant tissue and normal prostate and a marginal signal-to-noise ratio [105]. Modern 3Tesla MRI systems are under investigation for their utility in increasing signal-to-noise ratios for DWI. Early studies with this technology report a high sensitivity (94%) and specificity (91%) in the peripheral zone, and a similarly high sensitivity (90%) and specificity (84%) in the transition zone [108]. Future research will further elucidate the role of DWI in the diagnosis and staging of prostate cancer, although preliminary studies are very promising.

### 3.5. Superparamagnetic Nanoparticles

Another MRI-based technology currently under active investigation is the use of lymphotropic superparamagnetic nanoparticles as a contrast agent to detect small and otherwise undetectable nodal metastases. This material is injected intravenously and is taken up by macrophages in normal lymph nodes, creating a contrast between these nodes and cancerous nodes where the macrophages have been replaced by tumor cells [109]. The technology does not rely on the size or shape of the lymph node, criteria on which traditional radiographic staging is based, and is thus suspected to be more accurate in detecting metastases especially in normal-sized nodes. It has been proven to be effective in several other cancers [109]. Investigations into its use in prostate cancer are ongoing. Harisinghani et al. found that lymphotropic nanoparticle-enhanced MRI (LNMRI) can detect metastases in small lymph nodes that would be considered benign on CT or unenhanced MRI, and that metastatic nodes were found outside of the classical field of lymph node dissection in a significant number of patients [110]. This study evaluated 80 patients with LNMRI prior to either pelvic lymphadectomy or diagnosis of metastases with CT, and found that the addition of superparamagnetic nanoparticles increased sensitivity of MRI from 45.4% to 100% with a specificity of 95.7%. It has been suggested that this technology may be of particular utility in patients who have high risk of metastatic disease on the basis of standard staging nomograms and conventional MRI, both of which offer fairly high negative predictive values for the detection of metastatic disease [13].

This technology may also be useful in detecting recurrent cancer, as well as guiding targeted radiation therapy. A recent study reported that in 26 patients with recurrent prostate cancer after radical prostatectomy, all of whom were candidates for salvage radiation therapy, LNMRI detected positive nodes in 6 patients, none of whom had enlarged nodes on axial imaging [111]. Further studies are needed before this promising but experimental technology enters wide-spread use.

## 4. Computed Tomography

At this time, CT has very little role in the diagnosis of prostate cancer or in staging of known cancers in patients with a low clinical suspicion of metastatic disease [13]. CT detects nodal metastases based on size, and in general CT has a very low diagnostic yield in low-risk patients due to a low incidence of large nodal metastases in these patients. The primary role of CT in prostate cancer is in staging for patients with suspected metastatic disease, for which is has variable sensitivity and specificity [112, 113]. O'Dowd et al. [114] recommend that CT be used for high-risk patients with PSA > 20 ng/mL, Gleason score > 7, or at least clinical stage T3 disease. For the purpose of detecting pelvic lymph node metastases, the sensitivity has been reported from 25% [115] to 85% [116], but is generally approximately 36% [117], which is not sufficiently accurate to justify widespread use except for selected high-risk patients. Similarly, for diagnosing bone metastases, CT is inferior to other modalities such as bone scans and MRI, and should not be widely used [13].

## 5. Positron Emission Tomography

The theory behind positron emission tomography (PET) is that prostate cancer, having a high metabolic rate, will consume glucose through the glycolytic pathway, which is associated with higher glucose uptake. PET uses a radiolabeled analogue of glucose, typically fluorodeoxyglucose (FDG), as a tracer to measure the metabolic rate of the tissue, and attempts to identify cancerous lesions based on their increased metabolism. Despite initial enthusiasm for PET as a diagnostic modality for prostate cancer, studies have shown mediocre results, with suboptimal sensitivity [118, 119]. Effert et al. found that they were unable to differentiate between primary prostate cancer and benign prostatic hypertrophy with FDG PET [120], as both exhibit increased metabolism. This is thought to be due to a lower rate of growth and thus lower level of radiotracer uptake by prostate cancers than by other cancers in the body for which PET has been found to be more useful [121]. Moreover, as FDG is excreted by the kidneys, it tends to accumulate in the bladder and the prostatic urethra, thus masking uptake by the prostate [121]. Even when this was compensated for by diuresis with furosemide prior to PET scan [122] or continuous bladder irrigation, sensitivity was not sufficient to reliably detect prostate cancer or for the detection of lymph node metastases [123].

Other radiotracers with different properties are being investigated. Several studies have used ^ 11^Carbon- and ^18^Fluoride-based agents, including ^11^Carbon-methionine, ^18^F-Fluorocholine, ^11^Carbon-choline, and ^11^Carbon-acetate, as tracers for detecting prostate cancer. These tracers are radiolabeled amino acids which are concentrated in lesions with high protein synthesis rates, and can theoretically be used to identify cancerous lesions. They are used in the diagnosis of many different cancers, with sound clinical background, and are undergoing further testing for use in the primary diagnosis of prostate cancer, with limited utility at this point due to inadequate accuracy [72, 124–126]. Moreover, due to rapid decay of  ^11^Carbon, the use of these agents necessitates a local cyclotron, limiting its widespread availability.

Several studies have evaluated the use of PET for the staging of prostate cancer. Schiavina et al. [127] found that ^11^C-choline PET performed better than clinical nomograms for predicting nodal metastases, with a sensitivity and specificity of 60% and 98%. Results from a study by Husarik at al. [128] found more discouraging staging accuracy with ^18^F-FCH PET, with only one in five histopathologically-confirmed metastatic lymph nodes detected by imaging.

The most promising results with PET/CT are in the field of detecting recurrent disease after primary therapy for prostate cancer [129]. Rinnab et al. found first that ^11^C-choline was useful for targeted salvage lymph node dissection after treatment for prostate cancer [130], and then more recently that ^11^C-choline has a high sensitivity (93%) and PPV (80%) for detecting local recurrence or distant metastases [131]. This more recent study confirmed the results of several previous studies [132, 133] that PET/CT using ^11^C-choline may be a useful approach for detecting and localizing recurrent disease, but also showed that integrated PET/CT systems may be particularly helpful at low PSA levels.

The future of PET/CT in the field of prostate cancer is bright, with many developments on the horizon, from new tracers and technology to novel reading techniques. Li et al. recently reported that using a ratio of uptake values between prostate tissue and muscle as a primary measurement, ^11^C-choline PET achieved a sensitivity and specificity of 90% and 86%, may be a feasible technique to differentiate benign from malignant prostate lesions. Nuñez has proposed a combined use of FDG and ^11^C-methionine based on a temporal cascade of metabolic activity, with increased uptake of ^11^C-methionine in the early stages of cancer followed by relatively higher uptake of FDG during more advanced cancer stages [134]. Another group recently reported on the combined use of ^11^C-Choline and FDG for the detection of cancer after biochemical recurrence, and reported a sensitivity of 80% when a PSA cutoff of 1.9 ng/mL was used [135]. Hricak et al. suggested that novel iterative imagine reconstruction techniques will help to reduce artifact [13]. Several groups are also looking at new radiotracers that incorporate antibodies targeting molecules such as prostate-specific membrane antigen, which may significantly increase specificity of PET/CT [136, 137].

## 6. Radionuclide Scintigraphy

Prostascint is an immunoscintigraphic diagnostic mode utilizing [111] indium-capromab pendetide, a radiolabeled murine monoclonal antibody that is reactive with prostate-specific membrane antigen (PSMA), a glycoprotein expressed by prostate tissue. Images are captured with a SPECT gamma camera. It was approved by the FDA in 1996 for the detection of recurrent prostate cancer in soft tissue [138], with a reported sensitivity and specificity of 62% and 72% [139]. Seltzer et al. compared helical CT, PET, and Prostascint to evaluate for lymph node metastasis in patients with PSA recurrence. They found that Prostascint had a lower detection rate of metastatic disease than CT or PET [140]. Nagda et al. [141] performed a retrospective review of 58 patients who had rising PSA levels after prostatectomy but negative CT-scans and underwent a capromab pendetide scan. The PPV in detecting disease outside the prostate was 27%. The PPV for detecting prostatic fossa recurrence was 50%. Scan status was not found to be predictive of worsened biochemical recurrence free survival, indicating that decisions for adjuvant radiation therapy for biochemical recurrence should not be based upon the findings of the capromab study alone. Another study by Koontz et al. likewise did not find a difference in progression-free survival in patients with biochemical recurrences depending on findings of capromab scans. A major limitation of this technology is that the antibody targets an epitope that is only exposed by cancer cells that are dead or dying, which thus limits its sensitivity [142, 143]. The use of capromab studies varies from institution to institution, and more experience is needed before recommendations are solidified.

## 7. Conclusion

 We have described some of the recent advances in the field of imaging of prostate cancer, and highlighted some of the many new technologies on the horizon that will further enable rapid and effective diagnosis, staging, and follow-up of prostate cancer. With new modalities for visualizing prostate cancer, we are better than ever before able to characterize and localize lesions, and we are learning how to apply these capabilities to improved treatment decisions and more effective follow-up. These advances will hopefully contribute to improved long-term outcomes.
